# Seven-year retrospective study understanding the latent TB infection treatment cascade of care among adults in a low incidence country

**DOI:** 10.1186/s12889-021-10733-9

**Published:** 2021-05-21

**Authors:** K. Sullivan, C. Pease, A. Zwerling, R. Mallick, D. Van Dyk, S. Mulpuru, C. Allen, H. Alsdurf, G. G. Alvarez

**Affiliations:** 1 Ottawa Hospital, Department of Medicine, 501 Smyth Rd, Ottawa, K1H 8L6 Canada; 2 Ottawa University Faculty of Medicine, Ottawa, Canada; 3 Ottawa University School of Epidemiology and Public Health, Ottawa, Canada; 4 Ottawa Hospital Research Institute, Ottawa, Canada

**Keywords:** Latent tuberculosis infection, Cascade of care

## Abstract

**Background:**

Prevention of TB is paramount to achieving elimination targets as recommended by the World Health Organization’s action framework for low incidence countries striving to eliminate TB. Although the rates of TB in Canada are low, understanding the latent TB infection (LTBI) cascade is paramount to identifying gaps in care and treatment barriers, thereby increasing the effectiveness of preventive strategies. The purpose of this study was to examine the LTBI cascade of care and identify barriers to treatment completion in adults referred from primary care to a regional tertiary care TB clinic in Ottawa, Canada.

**Methods:**

Electronic medical records between January 2010 and December 2016 were reviewed retrospectively and an LTBI cascade of care was constructed from The Ottawa Hospital TB clinic and surrounding primary care clinics. A cohort of 2207 patients with untreated LTBI was used to ascertain the associations between demographic and clinical factors for both treatment non-initiation and non-completion using log-binomial univariable and multivariable regression models.

**Results:**

Of 2207 patients with untreated LTBI who were seen in the clinic during the study period, 1771 (80.2%) were offered treatment, 1203 (67.9% of those offered) started treatment, and 795 (66.1% of those started) completed treatment. In multivariable analysis, non-initiation of treatment was associated with older age (adjusted risk ratio [aRR] 1.06 per 5-year increase, 95% CI: 1.03–1.08) and female gender (aRR 1.28, 95% CI: 1.11–1.47). Non completion of treatment was associated with referral from the TB Clinic back to the primary care team following initial consult (aRR 1.62, 95% CI: 1.35–1.94) and treatment with the standard of 9 months of Isoniazid (9H) compared to 4 months of Rifampin (4R) (aRR 1.45, 95% CI:1.20–1.74).

**Conclusions:**

LTBI treatment completion was significantly decreased among patients who were referred back to primary care from the TB clinic. The 4R regimen resulted in more people completing LTBI treatment compared to 9H in keeping with a recently published RCT. Improved education, communication, and collaboration between tertiary care TB clinics and primary care teams may improve treatment completion rates and address the TB burden in low incidence communities in Canada.

**Supplementary Information:**

The online version contains supplementary material available at 10.1186/s12889-021-10733-9.

## Background

Tuberculosis (TB) remains one of the top 10 causes of death and the leading cause of death from a single infectious agent worldwide [1]. In 2018, the United Nations and World Health Organization (WHO) set forth a goal to end the TB epidemic by 2030 [2]. Despite efforts to reduce the burden of TB, 14.1 million individuals were treated for TB in 2018 and 2019 [1]. Perhaps more notably, the WHO estimates that 1.7 billion people have latent tuberculosis infection (LTBI) [3]. Individuals with LTBI have a 5 to 10% risk of developing active TB, furthering the spread of infection [4]. Prevention of TB is needed to reduce the disease burden and to achieve the WHO targets to end the TB epidemic by 2030 [2]. Interventions for TB prevention include infection control practices in health care facilities and congregate settings, vaccination of children with Bacille Calmette-Guerin (BCG) vaccine and, treatment of people with latent TB infection [2]. At the most recent United Nations high level meeting on TB in September of 2018 a target of 30 million people to be treated for LTBI in the next 5 years was set. Since the meeting, 6.3 million or 21% of the 5 year target of 30 million people have been started on TB preventive treatment in 2018 and 2019 [1]. In Canada, the incidence of LTBI is largely unknown; unlike active TB, there is no mandatory reporting of individuals with LTBI [5]. Although the incidence of TB in Canada is low, the rates of tuberculosis remain high in Canadian born Indigenous and foreign born individuals [5]. If Canada is to achieve TB elimination, more effort is required in the prevention of TB in these specific populations.

Current treatments available for the prevention of TB include isoniazid and/or shorter rifamycin based regimens [6]. These regimens have been shown to decrease progression from LTBI to active disease by 69–93% [7–9]. Many challenges exist for health care practitioners supporting their patients in completing LTBI treatment such as patients not wanting to take treatment because they do not feel sick; duration and adverse events of treatments, contra-indications to treatment and gaps in patient and provider knowledge about the importance of LTBI treatment. Furthermore, fewer physicians are managing TB cases on a regular basis because TB incidence remains low in Canada and many other developed countries, which may result in a decline in clinical expertise and quality of care [10]. In Québec, primary care physicians initiated only 26% of LTBI treatments between 1998 and 2007 [11]. One way to understand the reasons behind either not starting or not completing preventive treatment is to measure patient retention across sequential stages of care, known as a cascade of care analysis [12–14]. The present study aimed to document the LTBI cascade of care to identify treatment initiation and completion rates and the corresponding barriers to treatment completion in adults being referred to a regional referral outpatient TB clinic over a seven-year period in Ottawa, Canada.

## Methods

### Study population and design

A retrospective study of patients with untreated LTBI who attended the Ottawa Hospital TB clinic between January 1, 2010 and November 30, 2016 was undertaken. Individuals were determined to have LTBI based on a positive tuberculin skin test (TST) or interferon gamma release assay (IGRA), as per the Canadian TB Standards [15]. In the province of Ontario, the TST remains the standard of care for diagnosing LTBI primarily because it is free to patients. The IGRA is available but requires patients to pay $95.00 (CAN) out of pocket and thus is not frequently used. In certain cases, patients had both a TST and an IGRA. This was an individualized decision made with the patient and the physician. Individuals were excluded from the study if they had active TB, non-tuberculous mycobacteria disease (NTM) or were previously treated for active TB or LTBI. Electronic medical records of individual patients were reviewed to determine if a patient was offered treatment, started treatment and completed treatment. Treatment completion was defined as the proportion of individuals who completed LTBI treatment among those who initiated treatment. Additional definitions used in the study can be found in the Appendix. Ethics approval was obtained from the Ottawa Health Sciences Network Research Ethics Board. Informed consent was waived by the Ottawa Health Science Network Research Ethics Board.

### Study setting

The Ottawa TB clinic is located in a tertiary care center run by physicians that are specialists in Respirology, Infectious Diseases or Medical Microbiology. Individuals were referred to the TB clinic from primary care clinics throughout Eastern Ontario. If LTBI treatment was recommended, a monthly follow up visit was scheduled at the TB clinic until treatment completion. Patients obtained their medication from the pharmacy located at the hospital. In 2010, in order to shorten wait times for active TB cases, the TB clinic began sending patients back to the referring primary care team following the initial consultation with a detailed LTBI treatment plan for the primary care team (Appendix).

### Analysis

Descriptive statistics were used to summarize patient characteristics, the proportion of patients completing each step of the LTBI cascade and the reasons for losses at each step. The primary outcome was the proportion of patients that completed LTBI therapy among those who initiated treatment.

Log-binomial univariable and multivariable regression models were used to estimate risk ratios (RR) to assess associations between demographic and clinical risk factors and both treatment non-initiation and non-completion. Predefined clinically important variables included in the treatment non-initiation model were age, sex, reason for referral to the TB clinic, and year of consultation. Variables included in the treatment non-completion model were age, sex, reason for referral to the TB clinic, year of consultation, treatment regimen, and referral back to primary care team for treatment completion. Chi-square analysis was performed to assess the adverse events that resulted in treatment stoppage between regimens. Statistical analysis was carried out using SAS software, version 9.4 (SAS Institute Inc., Cary, NC, USA, 2017).

## Results

**Fig. 1:**
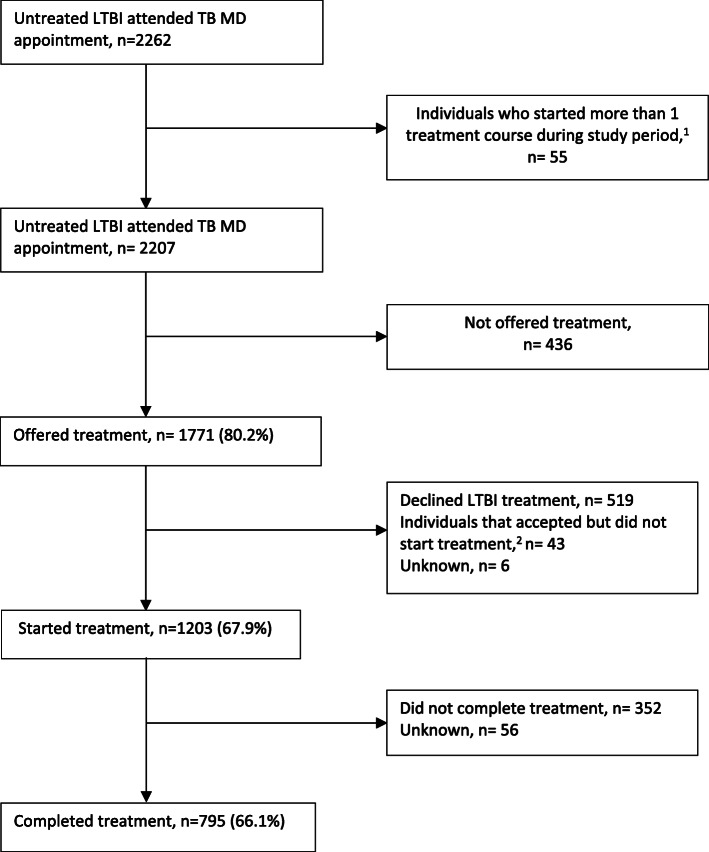
LTBI cascade of care at the Ottawa Hospital TB Clinic – January 1, 2010 to November 30, 2016. Percentages in the left-hand column represent the percentage of patients remaining in the cascade compared to the previous step in the cascade. ^1^Patients that started one treatment course during the study period and did not complete the course and started a second treatment course during the study period. ^2^Individuals who initially accepted treatment but never received a prescription and subsequently declined treatment or were lost to follow up

**Fig. 2:**
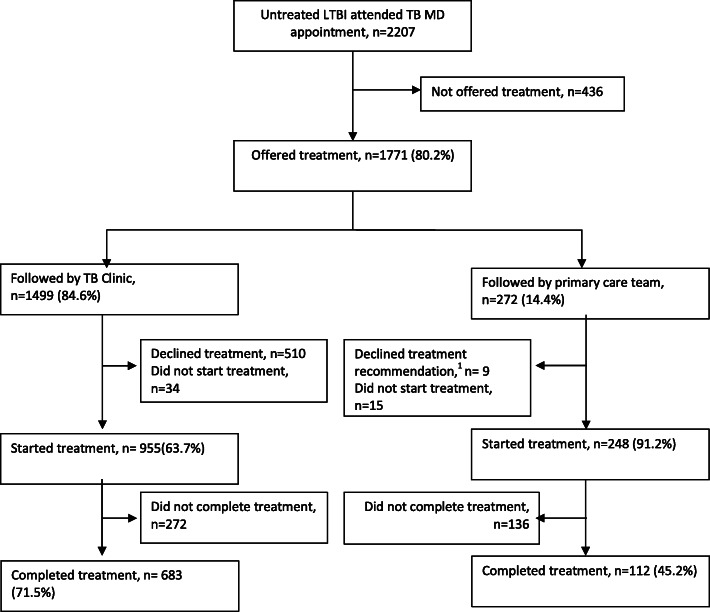
LTBI cascade of care at the Ottawa Hospital TB Clinic – January 1, 2010 to November 30, 2016 for the TB clinic and primary care clinics.Details about reasons for losses within the cascade of care are outlined in Table 2. Percentages represent the percentage of patients remaining in the cascade compared to the previous step in the cascade. ^1^The patient and the primary care team decided against treatment recommendations that were offered at the TB Clinic

**Fig. 3:**
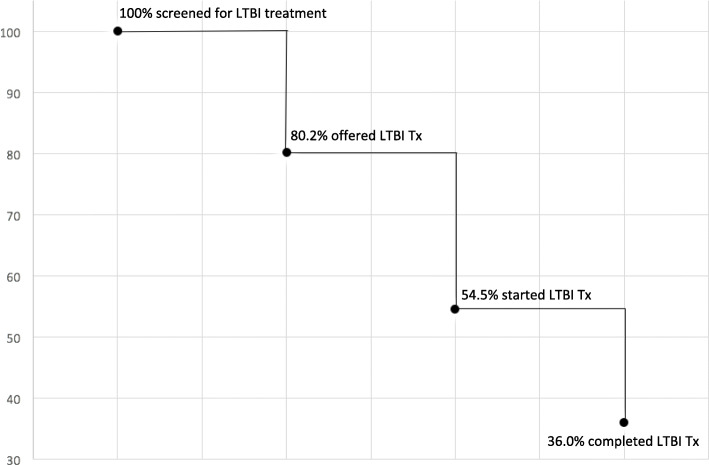
LTBI cascade of care at the Ottawa Hospital TB Clinic – January 1, 2010 to November 30, 2016.Percentages represent the percentage of patients remaining in the cascade compared the number of individuals screened for LTBI treatment

**Fig. 4:**
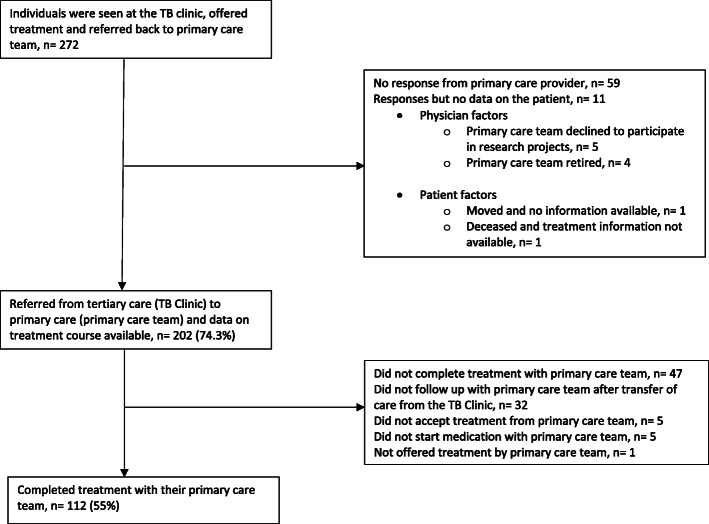
Information obtained from the primary care team on LTBI Treatment (*n* = 272).Information from the primary care team on individuals who were offered latent tuberculosis treatment at the Ottawa TB Clinic and subsequently followed for the duration of the treatment by their primary care team. Reasons treatment was not offered, not accepted, not started and not completed is captured in the Fig. 1

Between January 1, 2010 and November 30, 2016, data was collected on 2207 individuals who were referred to the TB Clinic at the Ottawa Hospital for LTBI assessment. Table 1 shows that 56.9% were female with a median age of 42 (IQR 30–53). The main reason for referral to the TB Clinic was for employment screening (735/2207). Overall, Fig. 1 shows that among those who started treatment, 66.1% (795/1203) completed treatment. Table 2 describes the main reasons for cascade attrition. Of those who were not offered treatment, 54.1% had discordant results between the TST and IGRA and 27.5% were lost to follow up. Of those who did not accept treatment, 48.4% did not specific a reason and 29.0% were lost to follow up. Of those who did not complete treatment, 50.7% were lost to follow up and 22.8% had an adverse event to the medication. Of those that started treatment 7.7% had an adverse event that led to treatment being stopped. Figure 2 shows that of the 955 patients who started treatment at the TB Clinic, 683 patients completed treatment (71.5%) at the TB Clinic. In comparison, of the 248 patients who started treatment with the TB clinic, 112 completed treatment (45.2%) with the primary care team. Figure 3 shows the cascade attrition from the initial consultation appointment, of those screened for LTBI treatment 36% completed treatment. Table 3 shows, of those prescribed isoniazid, 407/685 (59.4%) completed treatment and of those prescribed rifampin, 384/511 (75.1%) completed treatment. The proportion of adverse events which resulted in treatment being stopped with rifampin (4R, 27/511 = 5.3%) compared with isoniazid (9H, 64/685 = 9.3%) was significantly lower (*p*-value 0.012). Figure 4 shows significant attrition within the primary care LTBI treatment cascade with many patients not coming for follow up visits (*n* = 32) or completing treatment (*n* = 47).

**Table 1:** Demographic characteristics of patients (*n =* 2207 individuals) with consultation at the TB clinic between January 1, 2010 and November 30, 2016 by treatment location

	All patients (*n* = 2207)	Patients followed by TB Clinic (*n* = 1935)	Patients followed by Primary Care Team (*n* = 272)
Characteristics			
Males	951 (43.1%)	826 (42.7%)	125 (46.0%)
Females	1256 (56.9%)	1109 (57.3%)	147 (54.0%)
Age (years)^a^			
Median	42 (IQR 30-53)		
Mean (range)	42.5 (15-90)	42.6 (15-90)	42.1 (17-76)
<18	16 (0.7%)	15 (0.8%)	1 (0.4%)
18-65	2002 (90.7%)	1751 (90.5%)	251 (92.3%)
>65	189 (8.6%)	169 (8.7%)	20 (7.4%)
Reason for referral to TB Clinic			
Employment screening ^b^	735 (33.3%)	634 (32.8%)	101 (37.1%)
Rule out active TB disease^c^	576 (26.1%)	516 (26.7%)	60 (22.1%)
Contact tracing^d^	441 (20.0%)	388 (20.0%)	53 (19.5%)
Immunosuppressive therapy^e^	310 (14.0%)	255 (13.2%)	55 (20.2%)
Immigration screen^f^	145 (6.6%)	142 (7.3%)	3 (1.1%)

^a^Age of the individual at the initial consultation. ^b^Routine TB screening required for employment or Health Science students who required LTBI screening for their program (i.e. medical students and nursing students). ^c^Individuals referred by another physician to rule out active TB disease. ^d^Patient in contact with an active TB patient. ^e^Referred before starting immunosuppressive therapy including (i.e. prior to biologics, dialysis, chemotherapy or transplant). ^f^Referred following routine immigration screening

**Table 2:** Reason for losses within the Ottawa latent tuberculosis cascade of care (*n* = 1412) with comparison between patients followed at the TB Clinic and the Primary Care Team

Reason treatment not offered at the TB clinic			
	All Individuals (*n* = 436)	TB Clinic	Primary Care Team
Discordance^a^	236 (54.1%)	-	-
Lost to follow up^b^	120 (27.5%)	-	-
Age^c^	31 (7.1%)	-	-
Other^h^	26 (6.0%)	-	-
Did not specify^d^	12 (2.8%)	-	-
Comorbidities^c^	10 (2.3%)	-	-
Polypharmacy^c^	1 (0.2%)	-	-
Reason treatment not accepted			
	All Individuals (*n* = 568)	TB Clinic (*n* = 544)	Primary Care Team (*n* = 24)
Did not specify^d^	275 (48.4%)	274 (50.4%)	1 (4.2%)
Lost to follow up^b^	165 (29.0%)	163 (30.0%)	2 (8.3%)
Accepted but did not start treatment^i^	49 (8.6%)	34 (6.3%)	15 (62.5%)
Worried about side effects	19 (3.3%)	18 (3.3%)	1 (4.2%)
Discordance^a^	14 (2.5%)	14 (2.6%)	-
Does not want to take medications	11 (2.1%)	11 (2.0%)	-
Does not believe treatment is necessary	10 (1.9%)	8 (1.5%)	2 (8.3%)
Travelling	8 (1.4%)	8 (1.5%)	-
Drinking alcohol	6 (1.1%)	6 (1.1%)	-
Other^h^	6 (1.1%)	3 (0.6%)	3 (12.5%)
Treatment too long	4 (0.7%)	4 (0.7%)	-
Side effects + treatment too long	1 (0.2%)	1 (0.2%)	-
Reason treatment incomplete			
	All Individuals (*n* = 408)	TB Clinic (*n* = 272)	Primary Care Team (*n* = 136)
Lost to follow up^b^	207 (50.7%)	155 (57.0%)	52 (38.2%)
Adverse Event^f^	93 (22.8%)	84 (30.9%)	9 (6.6%)
Unknown^e^	56 (13.7%)	-	56 (41.2%)
Other^h^	28 (6.9%)	13 (4.8%)	15 (11%)
Did not specify^d^	10 (2.5%)	10 (3.7%)	-
Pregnancy	5 (1.1%)	4 (1.5%)	1 (0.7%)
Missed too many doses	4 (1.2%)	2 (0.7%)	2 (1.5%)
Change in health	3 (0.6%)	3 (1.1%)	-
Elected for another LTBI Tx^g^	1 (0.7%)	1 (0.4%)	-
Change in medication that interacts with LTBI treatment	1 (0.7%)	-	1 (0.7%)

^a^Patients with a positive TST and negative IGRA. ^b^Patients that: 1) required further baseline investigations prior to being offered treatment; 2) wanted another appointment prior to accepting treatment; 3) did not start treatment following accepting treatment; and 4) did not return to clinic for subsequent appointments. ^c^Risk of side effects with treatment is high given patient age, other medications, or comorbid medical conditions. ^d^The clinic note did not specify why the patient did not complete that step. ^e^Unknown if treatment started or completed due to no information received from the primary care team about the treatment course after leaving the TB Clinic. ^f^Adverse events that was felt to be treatment related and led to the LTBI treatment being stopped at the discretion of the TB physician or the patient. ^g^Patients who stopped their current treatment course in order to receive another regimen (i.e. patients that stopped isoniazid because they preferred to take rifampin). ^h^Other reason for losses within the cascade of care are outlined in the appendix.^i^Individuals who initially accepted treatment but never received a prescription and subsequently declined treatment or were lost to follow up

**Table 3:** Latent tuberculosis treatment completion rates stratified on reason for consultation at the TB clinic, gender and TB treatment selected for all individuals (*n* = 2207)

	Attended LTBI Consult (*n* = 2207)	Started Treatment (*n* = 1203)	Completed Treatment (*n* = 795)	% completed that started treatment
Gender				
Male	951	576	395	68.6%
Female	1256	627	400	63.8%
Reason for referral				
Employment and Health Science School Screen	735	351	224	63.8%
Rule out active TB	576	266	176	66.1%
Contact	441	277	178	64.3%
Immunosuppressive therapy	310	227	156	68.7%
Immigration Surveillance	145	82	61	74.4%
LTBI Treatment				
Isoniazid	-	685	407	59.4%
Rifampin	-	511	384	75.1%
Moxifloxacin	-	5	3	60.0%
Isoniazid and rifampin	-	1	1	100%
Unknown	-	1	0	0%

Table 4 shows that older age (adjusted risk ratio [aRR] 1.06 per 5-year increase, 95% CI: 1.03–1.08) and female sex (aRR 1.28, 95% CI: 1.11–1.47) were associated with treatment non-initiation in the adjusted multivariable model. Referral for contact tracing (aRR 0.71, 95% CI:0.59–0.86,) and referral prior to immunosuppressive therapy (aRR 0.30 95% CI (0.22–0.41)) compared to referral to rule out active tuberculosis were associated with increased initiation. When compared to the last year of consultation, several of the earlier years were associated with increased initiation. Table 5 shows that non completion of treatment was associated with referral from the TB Clinic back to the primary care team (aRR 1.62, 95% CI: 1.35–1.94) and with treatment with 9H compared to 4R (aRR 1.45, 95% CI:1.20–1.74).

**Table 4:** Risk ratios for non-initiation of treatment by demographic and clinical characteristics among individuals seen at the TB Clinic between January 2010 to December 2016. Risk ratios marked with an asterisk (*) are statistically significant. CI= confidence interval

Potential Risk Factors	Non-initiators/category total (%)	Unadjusted risk ratio (95% CI)	Adjusted risk ratio (95% CI) *n* = 568 patients^a^
Age, years (per 5-year increase)	-	1.03 (1.00-1.05)	1.06 (1.03-1.08)*
Sex			
Male	213/789 (27.0%)	Reference	Reference
Female	355/982 (36.2%)	1.34 (1.16-1.54)*	1.28 (1.11-1.47)*
Reason for referral			
Rule out active TB	180/446 (40.4%)	Reference	Reference
Employment screen	162/430 (37.7%)	0.93 (0.79-1.10)	0.90 (0.76-1.06)
Health science school screen	42/125 (33.6%)	0.83 (0.63-1.09)	0.84 (0.64-1.11)
Contact	106/383 (27.7%)	0.68 (0.56-0.85)*	0.71 (0.59-0.86)*
Immunosuppressive therapy	36/263 (13.7%)	0.34 (0.24-0.47)*	0.30 (0.22-0.41)*
Immigration surveillance	42/124 (33.9%)	0.84 (0.64-1.10)	0.88 (0.68-1.15)
Year of consultation			
2010	68/259 (26.3%)	0.64 (0.49-0.83)*	0.66 (0.52-0.85)*
2011	70/269 (26.0%)	0.64 (0.49-0.82)*	0.65 (0.51-0.84)*
2012	76/235 (32.3%)	0.79 (0.62-1.01)	0.80 (0.63-1.01)
2013	80/252 (31.7%)	0.78 (0.61-0.99)*	0.78 (0.62-0.98)*
2014	101/289 (34.9%)	0.85 (0.68-1.07)	0.86 (0.69-1.05)
2015	81/242 (33.5%)	0.82 (0.65-1.04)	0.88 (0.71-1.11)
2016	92/225 (40.9%)	Reference	Reference

^a^Model included age, sex, reason for referral to the TB clinic, and year of consultation

**Table 5:** Risk ratios for non-completion of treatment among individuals who started treatment for latent tuberculosis infection at the TB Clinic between January 2010 to December 2016. Risk ratios marked with an asterisk (*) are statistically significant. CI= confidence interval

Potential Risk Factors	Non-completion /category total (%)	Unadjusted risk ratio (95% CI)	Adjusted risk ratio (95% CI) *n* = 408 patients^a^
Age, years (per 5-year increase)	-	0.99 (0.97-1.02)	1.00 (0.97-1.03)
Sex			
Male	181/576 (31.4%)	Reference	Reference
Female	227/627 (36.2%	1.15 (0.98-1.35)	1.13 (0.97-1.32)
Reason for referral			
Rule out active TB	90/266 (33.8%)	Reference	Reference
Employment screen	100/268 (37.3%)	1.10 (0.88-1.39)	1.01 (0.81-1.26)
Health science school screen	27/83 (32.5%)	0.96 (0.67-1.37)	0.87 (0.62-1.22)
Contact	99/277 (35.7%)	1.06 (0.84-1.33)	1.00 (0.80-1.24)
Immunosuppressive therapy	71/227 (31.3%)	0.92 (0.71-1.19)	0.90 (0.70-1.16)
Immigration surveillance	21/82 (25.6%)	0.76 (0.50-1.13)	0.90 (0.60-1.35)
Year of consultation			
2010	49/191 (25.7%)	0.71 (0.51-0.99)*	0.84 (0.60-1.18)
2011	57/199 (28.6%)	0.79 (0.58-1.09)	0.91 (0.66-1.26)
2012	60/159 (37.7%)	1.04 (0.77-1.41)	1.08 (0.81-1.44)
2013	56/172 (32.6%)	0.90 (0.66-1.23)	0.90 (0.67-1.22)
2014	78/188 (41.5%)	1.15 (0.87-1.52)	1.10 (0.84-1.44)
2015	60/161 (37.2%)	1.03 (0.76-1.40)	1.02 (0.77-1.36)
2016	48/133 (36.1%)	Reference	Reference
Treatment regimen			
Isoniazid (9INH)	278/685 (40.6%)	Reference	Reference
Rifampin (4R)	127/511 (24.9%)	0.61 (0.51-0.73)*	0.69 (0.58-0.84)*
Moxifloxacin	2/5 (40.0%)	0.98 (0.33-2.89)	1.15 (0.38-3.45)
Referral back to primary care team	136/248 (54.8%)	1.92 (1.65-2.24)*	1.59 (1.33-1.91)*

^a^Model included age, sex, reason for referral to the TB clinic, year of consultation, treatment regimen, and referral back to primary care team for treatment completion

## Discussion

Our study shows that the transfer of patients on LTBI treatment back to primary care clinics was associated with a gap in the cascade that resulted in fewer patients completing treatment. In keeping with a large randomized controlled trial (RCT) [16], the study also confirmed that in practice completion of LTBI treatment is dependent on the regimen used and favours using shorter regimens such as 4R when compared to 9H in the Canadian setting. The treatment completion rate in our cascade of care of 66% of all patients that started treatment (1203/2207) is comparable to a meta-analysis which demonstrated that among those that started treatment 61% completed it (all regimens combined) [13]. In addition, factors associated with lower rates of treatment initiation were female sex and older age which are consistent with prior studies [17–20]. Older age is associated with increased adverse events with 9H which might explain the reluctance to initiate treatment.

Barriers to non-initiation of LTBI treatment were identified in our study. Strategies to address treatment non-initiation could include providing patients with educational resources prior to the initial consultation to increase understanding of LTBI and treatment. In addition, phone call reminders, expanding clinic hours, and offering telephone or virtual appointments may facilitate follow up and subsequent treatment for patients who cannot attend clinics in person.

The major barrier to completion of treatment identified in this study was the patient transfer back to the primary care team following TB clinic recommendations. The TB clinic had implemented a transfer policy to improve wait times to assess patients with active TB disease. Despite a detailed letter (Appendix) sent to primary care clinics to facilitate the transfer of care, completion rates in this group were poor compared to the TB clinic (45.2% compared to 71.5%, aRR 1.62, 95% CI: 1.35–1.94). Many patients were lost to follow up before ever seeing their primary care provider to continue LTBI treatment. Losses occurring at this level of the cascade point to the need for programmatic changes such as reminder emails or phone calls from the clinic to the patient, reminder emails to the primary care teams to arrange follow up, and possibly expanded clinic times or virtual appointments to accommodate for patient follow up appointments [21]. As TB rates continue to decrease across Canada and other low TB burden countries, there is less TB expertise among clinicians. A Canadian study done in Ontario demonstrated that physician experience was associated with a decrease in all-cause mortality among patients being treated for active TB disease [10]. Furthermore, a study between 1998 and 2007, showed that TB patients that started treatment with a primary care physician were less likely to complete treatment when compared to specialist clinics in the province of Québec [11]. TB education sessions to refresh primary care providers’ knowledge about newer, shorter regimens may assist in improving completion rates. In addition, mandatory reporting of the diagnosis of LTBI to local public health departments could improve the completion rates by allowing public health teams to follow up with primary care clinics and patients. As a result of this study, our clinic has stopped referring patients back to primary care during the treatment course until we can develop a better system to ensure completion however this will inevitably drive up wait times for active TB cases. Further study is needed to develop strategies to address this important gap, since primary care has a central role to play in the prevention and elimination of TB in Canada.

Another major barrier to treatment completion identified in this study was the choice of treatment regimen. The two main regimens offered at this clinic during the study period were rifampin for 4 months (4R) and isoniazid for 9 months (9H). An open-label non-inferiority RCT comparing 4R and 9H showed increased completion rates in individuals prescribed 4R (78.8%) compared to those prescribed 9H (63.2%) [16]. Our study demonstrated comparable completion rates (75% completion with 4R). Similarly to this RCT, our study showed that adverse events that resulted in treatment non-completion were significantly less with 4R compared to 9H. Although, the baseline proportion of adverse events leading to non-completion in our study were higher than in the published RCT (4R, 27/511, 5.3% vs 9H, 64/685, 9.3%, *p* value = 0.012 compared to 4R, 2.6% vs 9H, 5.4% in the RCT) [16]. This finding likely reflects clinical practice before the publication of the 2018 study by Menzies et al. and may have occurred because the clinicians using 4R did not have as much experience using the regimen. Overall, shorter regimens have been shown to increase completion rates [8], which highlights an opportunity to implement even shorter rifamycin based regimens including once-weekly isoniazid-rifapentine 3 months regimen (3HP) or daily isoniazid-rifapentine for 1 month (1HP) to further increase completion rates [22].

Of note, 33.3% of LTBI patients were referred following employment screening likely due to referral of healthcare workers following routine screening undertaken for all employees at our large tertiary care centre during the study period. Recent data suggest a low risk of reactivation amongst TST positive healthcare workers [23, 24]. Given this, there has since been a shift in our hospital policy such that annual LTBI screening is reserved for high-risk individuals. However, this policy change occurred after the study period and so is not reflected in our data.

Strengths of this study include that the Ottawa TB clinic reflects the real-world experience of many other specialized TB clinics in large, urban centers. A long timeframe for analysis (seven years) among a large population of individuals at a specialized tertiary care clinic in a low burden country allowed for detailed analysis of the LTBI treatment cascade of care. The retrospective design of this study limits the ability to understand the reasons the decisions made by clinicians and patients, especially when lost to follow up. Data collection was limited to electronic chart review based on clinician documentation. We did not have consistent information on other possible confounders such as socioeconomic status, comorbidities and also the experience of the primary care teams in treating LTBI. Treatment adherence was assessed based on patient follow up at the TB Clinic and not by number of doses ingested however this does represent standard practice in a low burden TB setting. Information on treatment course for patients referred to their primary care team after being offered treatment at the TB Clinic was available for 74% of patients.

## Conclusions

Effective treatment of LTBI is a central pillar in lowering the rates of TB worldwide as we move towards TB elimination. In low incidence countries like Canada examining the LTBI cascade to improve completion of TB prevention treatments can assist with this goal. In this study, transfer of patients on LTBI treatment back to the primary care clinics was identified as leading to a major gap in the cascade that resulted in decreased rates of treatment completion. Primary care has a central role to play in the prevention of TB and further research is needed to look at ways of expanding its role in the context of specialty TB clinics, particularly in urban centers in Canada. Use of shorter rifamycin based regimens like 4R improved the number of people who completed LTBI treatment.

## Supplementary Information

**Additional file 1.** Supplementary information on Design and Definitions.

## Data Availability

The datasets used and/or analyzed during the current study are available from the corresponding author upon reasonable request.

## Abbreviations

TB: Tuberculosis
LTBI: Latent tuberculosis infection
9H: 9 months of Isoniazid
4R: 4 months of Rifampin

## References

[1] Organization WH (2020). Global Tuberculosis Report.

[2] Organization GWH. Global Tuberculosis Report 2019. Executive Summary. 2019 17 October 2019. Contract No.: WHO/CDS/TB/2019.15.

[3] Houben RM, Dodd PJ (2016). The global burden of latent tuberculosis infection: a re-estimation using mathematical Modelling. PLoS Med.

[4] The Lancet Respiratory M (2016). Focus on latent tuberculosis. Lancet Respir Med.

[5] LaFreniere M, Hussain H, He N, McGuire M (2019). Tuberculosis in Canada: 2017. Can Commun Dis Rep.

[6] Getahun H, Matteelli A, Chaisson RE, Raviglione M (2015). Latent mycobacterium tuberculosis infection. N Engl J Med.

[7] International Union Against Tuberculosis Committee on Prophylaxis (1982). Efficacy of various durations of isoniazid preventive therapy for tuberculosis: five years of follow-up in the IUAT trial. International Union Against Tuberculosis Committee on Prophylaxis. Bull World Health Organ.

[8] Pease C, Hutton B, Yazdi F, Wolfe D, Hamel C, Quach P (2017). Efficacy and completion rates of rifapentine and isoniazid (3HP) compared to other treatment regimens for latent tuberculosis infection: a systematic review with network meta-analyses. BMC Infect Dis.

[9] Sterling TR, Villarino ME, Borisov AS, Shang N, Gordin F, Bliven-Sizemore E (2011). Three months of rifapentine and isoniazid for latent tuberculosis infection. N Engl J Med.

[10] Khan K, Campbell A, Wallington T, Gardam M (2006). The impact of physician training and experience on the survival of patients with active tuberculosis. CMAJ..

[11] Rubinowicz A, Bartlett G, MacGibbon B, Greenaway C, Ronald L, Munoz M (2014). Evaluating the role of primary care physicians in the treatment of latent tuberculosis: a population study. Int J Tuberc Lung Dis.

[12] Reid MJ, Goosby E (2017). Lessons learned from the HIV care cascade can help end TB. Int J Tuberc Lung Dis..

[13] Alsdurf H, Hill PC, Matteelli A, Getahun H, Menzies D (2016). The cascade of care in diagnosis and treatment of latent tuberculosis infection: a systematic review and meta-analysis. Lancet Infect Dis.

[14] Subbaraman R, Nathavitharana RR, Mayer KH, Satyanarayana S, Chadha VK, Arinaminpathy N (2019). Constructing care cascades for active tuberculosis: a strategy for program monitoring and identifying gaps in quality of care. PLoS Med.

[15] Gale-Rowe M, Menzies D, Sutherland J, Wong T (2014). Chapter A. Highlights of the new 7th edition of the Canadian tuberculosis standards. Can Commun Dis Rep.

[16] Menzies D, Adjobimey M, Ruslami R, Trajman A, Sow O, Kim H (2018). Four months of rifampin or nine months of isoniazid for latent tuberculosis in adults. N Engl J Med.

[17] Gershon AS, McGeer A, Bayoumi AM, Raboud J, Yang J (2004). Health care workers and the initiation of treatment for latent tuberculosis infection. Clin Infect Dis.

[18] Horsburgh CR, Goldberg S, Bethel J, Chen S, Colson PW, Hirsch-Moverman Y (2010). Latent TB infection treatment acceptance and completion in the United States and Canada. Chest..

[19] Colson PW, Hirsch-Moverman Y, Bethel J, Vempaty P, Salcedo K, Wall K (2013). Acceptance of treatment for latent tuberculosis infection: prospective cohort study in the United States and Canada. Int J Tuberc Lung Dis..

[20] Swift MD, Molella RG, Vaughn AIS, Breeher LE, Newcomb RD, Abdellatif S (2020). Determinants of latent tuberculosis treatment acceptance and completion in healthcare personnel. Clin Infect Dis.

[21] Liu Q, Abba K, Alejandria MM, Sinclair D, Balanag VM, Lansang MA (2014). Reminder systems to improve patient adherence to tuberculosis clinic appointments for diagnosis and treatment. Cochrane Database Syst Rev.

[22] Swindells S, Ramchandani R, Gupta A, Benson CA, Leon-Cruz J, Mwelase N (2019). One month of Rifapentine plus isoniazid to prevent HIV-related tuberculosis. N Engl J Med.

[23] Sosa LE, Njie GJ, Lobato MN, Bamrah Morris S, Buchta W, Casey ML (2019). Tuberculosis screening, testing, and treatment of U.S. health care personnel: recommendations from the National Tuberculosis Controllers Association and CDC, 2019. MMWR Morb Mortal Wkly Rep.

[24] Sester M, van Crevel R, Leth F, Lange C (2015). Numbers needed to treat to prevent tuberculosis. Eur Respir J.

