# Supplementary material for: Seven-year retrospective study understanding the latent TB infection treatment cascade of care among adults in a low incidence country
Source: BMC Public Health. 2021 May 21;21:964. doi: 10.1186/s12889-021-10733-9 (PMC8140464; doi:10.1186/s12889-021-10733-9)
Supplement: Supplementary file 1 — Additional file 1. Supplementary information on Design and Definitions. [file 12889_2021_10733_MOESM1_ESM.docx]

**APPENDIX/SUPPLEMENT**

Supplementary information on Design and Definitions

**Design**

Secondary variables collected included patient demographics, the reason for referral to the TB clinic, TST and/or IGRA result, date of LTBI diagnosis, date of first appointment at the TB clinic, reason(s) physician did not offer treatment, reason(s) patient declined treatment, the type of treatment selected, the length of treatment and the reason for not completing treatment, if applicable. Information on treatment completion was obtained from the primary care team by a fax request. If no response was received from the fax the primary care teams were called by phone.

**Definitions**

Individuals were determined to have LTBI based on a positive TST or IGRA, as per the Canadian TB Standards(1) . In the province of Ontario, the tuberculin skin test (TST) remains the standard of care for diagnosing LTBI primarily because it is free to patients. The interferon-gamma release assay (IGRA) is available but requires patients to pay $95.00 (CAN) out of pocket and thus is not frequently used. Patients were offered treatment by the physician at the TB clinic as reflected in the electronic medical record. Treatment acceptance was defined as the patient agreeing to take treatment for LTBI as reflected in the electronic medial record. Receiving a prescription for LTBI medication from a TB physician was used as a surrogate indicator of the patient starting LTBI treatment. There are no universally accepted treatment adherence and completion definitions (2). In this study, attendance to monthly follow up appointments at the TB Clinic was used as a surrogate for treatment adherence. Treatment was considered complete if the individual completed 90% of the required total months of treatment; or had a note in the chart stating treatment had been completed. Completion by months on treatment was based on the difference between the appointment date where the individual received a prescription for medication and the final appointment that the individual had at the TB Clinic. Adverse events were considered only for those individuals that stopped treatment as a result.

**References**

1. Menzies D, Alvarez, G.G., Khan K. Canadian Tuberculosis Standards, 7th Edition, Treatment of Latent Tuberculosis Infection. In: Public Health Agency of Canada, editor. 2014.

2. Pease C, Hutton B, Yazdi F, Wolfe D, Hamel C, Quach P, et al. Efficacy and completion rates of rifapentine and isoniazid (3HP) compared to other treatment regimens for latent tuberculosis infection: a systematic review with network meta-analyses. BMC Infect Dis. 2017;17(1):265.

**Transfer of care from the TB Clinic to Primary Care Teams**

In order to shorten wait times, the TB clinic began sending patients back to the referring community primary care team following the initial consultation, where the TB team made recommendations whether or not the patient should initiate LTBI treatment and set up a detailed treatment plan for the primary care team in the form of a letter outlined below. Patients were referred back to the primary care team beginning in 2010 through to 2016.

Information on treatment completion was obtained from the primary care team by a fax request. An initial 2 page fax was sent to the primary care team. The first page explained the study and the information that was being requested. The second page had the name of the patient demographics and a form to complete on the treatment information to be faxed back. If no response was received in 1 week a phone call was made to the primary care clinics and a follow up fax was sent. If no response was received, a second phone call reminder was done the following week.

**Transfer of care letter sent from the TB Clinic to the Primary Care Team**

**Fax request for treatment information sent to the Primary Care Team**


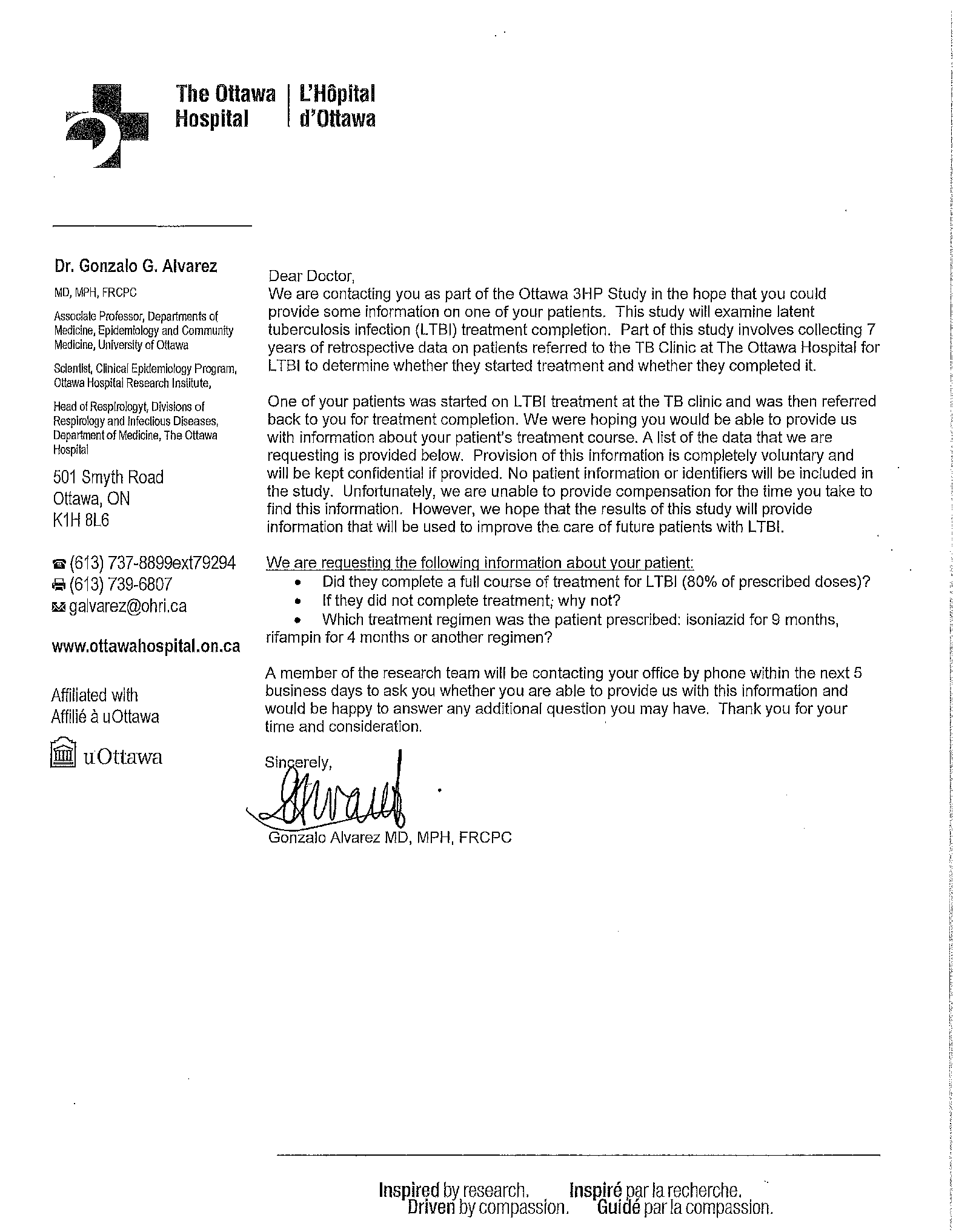

**Fig. 1** Latent tuberculosis cascade of care at the Ottawa Hospital TB Clinic between January 1, 2010 and November 30, 2016 based on treatment type.

Blue = isoniazid, orange = rifampin, green = other (moxifloxacin, isoniazid and rifampin, unknown)

**Table 1** Other reasons for losses within the Ottawa latent tuberculosis cascade of care with comparison between patients followed at the TB Clinic and the Primary Care Team

| Reason treatment not offered | | | |
| --- | --- | --- | --- |
|  | All Individuals (n= 26) | TB Clinic | Primary Care Team |
| MDRTB contact^1^ | 5 | - | - |
| Moved^2^ | 3 | - | - |
| Compliance concerns^3^ | 2 | - | - |
| Pregnancy^4^ | 9 | - | - |
| Physician discretion^5^ | 7 | - | - |
| Reason treatment not accepted | | | |
|  | All Individuals (n= 8) | TB Clinic (n= 4) | Primary Care Team (n= 4) |
| Moved^2^ | 2 | 2 |  |
| Decided against recommendation ^6^ | 2 | 1 | 1 |
| Accepted but did not start treatment^8^ | 2 | 1 | 1 |
| Discordance^7^ | 1 | - | 1 |
| No information received from primary care team | 1 | - | 1 |
| Reason treatment incomplete | | | |
|  | All Individuals (n= 28) | TB Clinic (n= 13) | Primary Care Team (n= 15) |
| Discordance^7^ | 2 | 2 |  |
| Moved^2^ | 7 | 6 | 1 |
| Patient does not want to take medications | 5 | 5 |  |
| No documentation on treatment completion from primary care team | 6 | - | 6 |
| Completion with primary care team did not meet 80% completion definition for our study | 8 | - | 8 |

^1^Patient was in contact with a case of multi-drug resistant tuberculosis. ^2^Moved from Ottawa. ^3^Concerns about patient compliance to treatment regimen based on the discretion of the physician. ^4^ Female participants that were pregnant or planning to become pregnant. ^5^ Risk of taking treatment outweighed the benefit based on physician discretion. ^6^ The patient and a physician outside of the TB Clinic decided against treatment recommendations. ^7^ Negative IGRA after a positive TST. ^8^Individuals who initially accepted treatment but never received a prescription and subsequently declined treatment or were lost to follow up

**Table 2.** Latent tuberculosis treatment completion rates based on reason for consultation at the TB clinic, gender and TB treatment selected for individuals followed at the TB Clinic for total treatment course (n= 1935)

|  | Attended LTBI Consult (n= 1935) | Started Treatment  (n= 955) | Completed Treatment  (n= 683) | % completed that started treatment |
| --- | --- | --- | --- | --- |
| Reason for referral |  |  |  |  |
| Employment and Health Science School Screen | 634 (32.8%) | 261 | 187 | 71.6% |
| Rule out active TB | 516 (26.4%) | 213 | 153 | 71.8% |
| Contact | 388 (26.7%) | 228 | 151 | 66.2% |
| Immunosuppressive therapy | 255 (13.2%) | 173 | 131 | 75.7% |
| Immigration Surveillance | 142 (7.3%) | 80 | 61 | 76.2% |
| Gender |  |  |  |  |
| Male | 826 | 462 | 342 | 74.0% |
| Female | 1109 | 493 | 341 | 69.1% |
| LTBI Treatment |  |  |  |  |
| Isoniazid | - | 485 | 319 | 65.8% |
| Rifampin | - | 464 | 360 | 77.6% |
| Moxifloxacin | - | 5 | 3 | 60.0% |
| Isoniazid and rifampin | - | 1 | 1 | 100% |

**Table 3.** Latent tuberculosis treatment completion rates based on reason for consultation at the TB clinic, gender and TB treatment selected for individuals offered treatment at the TB Clinic and followed for treatment with the primary care team (n= 248)

|  | Attended LTBI Consult | Started Treatment | Completed Treatment | % completed that started treatment |
| --- | --- | --- | --- | --- |
| Reason for referral |  |  |  |  |
| Employment and Health Science School Screen | 101 (37.1%) | 90 | 37 | 41.1% |
| Rule out active TB | 60 (22.1%) | 53 | 23 | 43.4% |
| Contact | 53 (19.5%) | 49 | 27 | 55.1% |
| Immunosuppressive therapy | 55 (20.2%) | 54 | 25 | 44.4% |
| Immigration Surveillance | 3 (1.1%) | 2 | 0 | 0.0% |
| Gender |  |  |  |  |
| Male | 125 | 114 | 53 | 46.5% |
| Female | 147 | 134 | 59 | 44.0% |
| LTBI Treatment |  |  |  |  |
| Isoniazid | - | 200 | 88 | 44.0% |
| Rifampin | - | 47 | 24 | 51.1% |
| Unknown | - | 1 | 0 | 100% |
